# The Role of the PAX8/PPAR*γ* Fusion Oncogene in Thyroid Cancer

**DOI:** 10.1155/2008/672829

**Published:** 2008-10-29

**Authors:** Kimberly A. Placzkowski, Honey V. Reddi, Stefan K. G. Grebe, Norman L. Eberhardt, Bryan McIver

**Affiliations:** ^1^Division of Endocrinology, Department of Medicine, Mayo Clinic & Foundation, Rochester, MN 55905, USA; ^2^Department of Laboratory Medicine and Pathology, Mayo Clinic & Foundation, Rochester, MN 55905, USA; ^3^Department of Biochemistry and Molecular Biology, Mayo Clinic & Foundation, Rochester, MN 55905, USA

## Abstract

Thyroid cancer is uncommon and exhibits relatively low mortality rates. However, a subset of patients experience inexorable growth, metastatic spread, and mortality. Unfortunately, for these patients, there have been few significant advances in treatment during the last 50 years. While substantial advances have been made in recent years about the molecular genetic events underlying papillary thyroid cancer, the more aggressive follicular thyroid cancer remains poorly understood. The recent discovery of the PAX8/PPAR*γ* translocation in follicular thyroid carcinoma has promoted progress in the role of PPAR*γ* as a tumor suppressor and potential therapeutic target. The PAX8/PPAR*γ* fusion gene appears to be an oncogene. It is most often expressed in follicular carcinomas and exerts a dominant-negative effect on wild-type PPAR*γ*, and stimulates transcription of PAX8-responsive promoters. PPAR*γ* agonists have shown promising results in vitro, although very few studies have been conducted to assess the clinical impact of these agents.

## 1. INTRODUCTION

Thyroid cancer is the most frequent endocrine malignancy. While relatively uncommon, the American Cancer Society estimates that there will be over 37 000 new cases in 2008 in the United States [1]. The majority of thyroid cancers are well-differentiated malignancies originating from the thyroid follicular cells. Papillary thyroid carcinomas (PTCs) are the most frequent histotype, particularly in iodine-sufficient areas, while follicular thyroid carcinoma (FTC) and Hürthle cell carcinomas (HCCs) together represent around 20% of thyroid cancer. However, these latter thyroid cancer types are often more aggressive, more advanced at the time of diagnosis, less responsive to traditional therapy, and more likely to cause both morbidity and mortality. FTC share similar cytologic features with follicular adenomas (FAs) and are distinguished only by the presence of invasion beyond the tumor capsule or into blood vessels on pathology. This common histology suggests an adenoma to carcinoma sequence, with adenomas representing a premalignant lesion, though this remains unproven.

However, the peroxisome proliferator-activated receptors (PPARs), including *α*, *β*, *δ*, and *γ* subtypes, are part of the ubiquitous nuclear hormone receptor superfamily that has been the focus of considerable research over the last two decades. PPAR*γ* is best known in the endocrine world for its role in adipogenesis and insulin sensitization. However, it also plays a role in cell cycle control, inflammation, atherosclerosis, apoptosis, and carcinogenesis through its influence on gene expression involving multiple cell signaling pathways [2]. Research on PPAR*γ* as a potential thyroid proto-oncogene was accelerated by the discovery of a chromosomal translocation involving the PPAR*γ*-1 gene in a subset of follicular carcinomas [3]. Since that time, considerable effort has gone into further clarifying the possible effect of PPAR*γ* in several cancers, including FTC.

## 2. PPAR*γ* AND THYROID CANCER

Chromosome analysis and microsatellite mapping techniques have revealed a number of chromosomal changes associated with thyroid carcinogenesis, and pointed to specific loci that might harbor oncogenes or tumor suppressor genes, including a region spanning the fragile site on chromosome 3p14, through 3pter that is often disrupted in follicular thyroid cancers [4]. Jenkins et al. had already described a somatic mutation involving 3p25-p21 region in three cases of follicular thyroid cancer [5, 6], and there followed a number of reports of balanced translocations or aneusomy involving this chromosomal region in FTC, HCC, PTC, and anaplastic thyroid cancers (ATCs) [7–11]. A specific translocation, t(2;3)(q13;p25), was described in a case of aggressive FTC associated with bone metastases [11], though it was also seen in some FAs [10]. The importance of this chromosomal rearrangement and the mechanism of its oncogenic activity remained unclear until Kroll et al. mapped the involved 3p25 locus in a number of FTC to the PPAR*γ* gene in 2000 [3]. Thus these data were the first to convincingly link the PPAR*γ* gene to thyroid cancer. Altered PPAR*γ* activity has subsequently been shown to have a potential role in several types of thyroid cancer.

Several reports of ATC, FTC, and some PTC cell lines have demonstrated PPAR*γ* mRNA expression by reverse-transcription polymerase chain reaction (RT-PCR), without identifiable PPAR*γ* mutations or translocations [12–15]. In these PPAR*γ*-positive cell lines, PPAR*γ* ligands (troglitazone [15, 16], ciglitazone [12, 14, 17], rosiglitazone [12], prostaglandin J2 [13, 15, 17], and RS1303 [13]) inhibit growth of cells, while no change in growth is seen in PPAR*γ*-negative cell lines. Growth suppression was dose-dependent [13, 16, 17], and one study found a correlation between PPAR*γ* expression and response to PPAR*γ* ligands [13], though another did not [16]. Levels of Bax protein and c-myc, both apoptosis-related proteins, were increased in a dose-dependent fashion by treatment with PPAR*γ* ligand [13, 15], although one study found increased levels of a different apoptotic protein, Bcl-2, rather than of Bax [12]. Inhibition of cell invasion [13], attachment [12, 17], and anchorage-independent growth on soft agar [12], all features of malignancy, are also seen with PPAR*γ* ligand treatment in thyroid cancer cell lines. Finally, evidence of cell death, with decreased viable cell numbers and increased rates of necrosis and apoptosis, have been reported by some groups following PPAR*γ* agonist treatment [12, 17]. This reduction in cell viability may be inhibited by coincubation with GW9662, a selective PPAR*γ* antagonist, suggesting that the described changes in cell growth and survival were truly PPAR*γ*-dependent [12].

Addition of PPAR*γ* agonist to PPAR*γ*-positive cells lines led to an increased portion of cells in G_0_/G_1_ with a reduction of cells in G_2_/M and S phase, consistent with decreased cell proliferation [12]. DNA synthesis appeared to be slowed with decreased ^3^H-thymidine incorporation in these cells, while expression of the cell-cycle progression inhibitors p21^cip1^ and p27^kip1^ were increased [12]. Overexpression of the PPAR*γ* gene by transfection into PPAR*γ*-positive or -negative cell lines similarly decreased colony formation and triggered nuclear condensation, fragmentation of chromatin and apoptosis, with G_0_/G_1_ cell cycle arrest [12, 14–16]. Together, these data provide strong support that PPAR*γ* has a tumor suppressive effect in thyroid follicular cells, which is consistent with results in other nonthyroid cell lines [18–23].

One of the few available animal models of follicular thyroid carcinoma was created by generating homozygous mutations in the thyroid receptor gene TR*β*, a mutation initially described in a patient with thyroid hormone resistance syndrome [24] and referred to as the PV/PV mutant. Homozygous TR*β* ^PV/PV^ mice develop follicular thyroid cancer with predictable progression from thyroid hyperplasia to capsular and vascular invasion, more extensive soft tissue invasion and ultimately lung metastasis at an early age [25]. Analysis of gene activity by cDNA microarray analysis in TR*β* ^PV/PV^ mice demonstrates altered regulation of several genes compared to wild-type siblings. Many of these genes are implicated in tumor-formation, metastasis, invasion, cell cycle control and apoptosis. PPAR*γ*-mediated pathways, however, are downregulated in these mice, hinting at a role for PPAR*γ* inhibition in this thyroid cancer model [26].

Early in the histologic progression of thyroid disease in this TR*β* ^PV/PV^ mouse model expression of PPAR*γ* mRNA, assessed by Northern blot analysis, was diminished approximately 50% compared to wild-type siblings. Furthermore, not only was PPAR*γ* activity suppressed, but the mRNA activity in mutant mice did not increase with age as was seen in their wild-type counterparts [27]. Data from humans has also implicated a functional downregulation of PPAR*γ* expression in human thyroid tumors that did not carry PPAR*γ* translocations, in studies using semiquantitative PCR, real-time RT-PCR, and microarray analyses [28, 29]. Furthermore, tumors with reduced PPAR*γ* expression showed an increased incidence of distant metastases, local invasion, and areas of poor differentiation [28]. These findings suggest that downregulation of wild-type PPAR*γ* may be a key event in thyroid carcinogenesis.

Homozygous PPAR*γ* ^+/−^ mice, incorporating a loss of function mutation in one allele of the PPAR*γ* gene, have been used as an animal model to evaluate the molecular genetic events ultimately leading to carcinogenesis in colon, breast, and ovarian tumors [30, 31]. Deletion of both alleles of the PPAR*γ* gene, however, is universally lethal to embryos. In thyroid cancer, the TR*β* ^PV/PV^ mouse model was used to further elucidate the mechanism of PPAR*γ* tumorigenesis. TR*β* ^PV/PV^ mice were crossed with PPAR*γ* ^+/−^ mice to obtain TR*β* ^PV/PV^PPAR*γ* ^+/−^ offspring. In these mice, PPAR*γ* mRNA and lipoprotein lipase (LpL) expression were reduced compared to wild-type mice. LpL is a downstream target gene for PPAR*γ*, thereby confirming diminished PPAR*γ* action. Furthermore, LpL mRNA and protein expression were further reduced in the mutant TR*β* ^PV/PV^PPAR*γ* ^+/−^ mice compared to both wild-type and TR*β* ^+/+^PPAR*γ* ^+/−^ mice [32].

Western blot analysis demonstrated activation of the NF*κ*B pathway in these TR*β* ^PV/PV^PPAR*γ* ^+/−^ mice [32], which is consistent with data in the PPAR*γ* ^+/−^ mouse model [33]. Expression of cyclin D1, a cell cycle regulator important in the progression from G_1_to S phase and a downstream target of NF*κ*B, was significantly increased. Also, cell cycle analysis in TR*β* ^PV/PV^PPAR*γ* ^+/−^ mice showed a shortened G_0_/G_1_ phase and decreased apoptosis compared to TR*β* ^PV/PV^PPAR*γ* ^+/+^ mice [32]. Cyclin D1 is known to be overexpressed in human thyroid carcinomas [34] and in vitro addition of a PPAR*γ* agonist to human ATC cell lines suppresses cyclin D1 levels [12], confirming that this effect is related to alterations in PPAR*γ* rather than TR*β*. Caspase-3, a gene critical in the apoptotic signal cascade, was significantly reduced in TR*β* ^PV/PV^PPAR*γ* ^+/−^ mice, a finding consistent with previous data on the downstream effects of NF*κ*B activation in prostate cancer cells [35]. Conversely, increased levels of caspase-3 activation were seen in vitro with PPAR*γ* agonist therapy of FTC [17] and ATC cell lines [12]. Importantly, rosiglitazone therapy slowed tumor growth and reduced capsular invasion in TR*β* ^PV/PV^ mice, suggesting that activation of the PPAR*γ* pathway delays disease progression [32].

In summary, several lines of evidence, in human tumors, cell lines, and animal models strongly support the hypothesis that PPAR*γ* inhibition, downregulation, or insufficiency appears to be tumorigenic in the thyroid. This appears to be mediated through PPAR*γ* effects on cell cycle progression and inhibition of apoptosis, thereby contributing to tumor development or progression.

## 3. PAX8/PPAR*γ* GENE TRANSLOCATION AND THYROID CANCER

Following the identification of a frequent translocation in follicular thyroid cancer, involving 3p25 and 2q13, Kroll confirmed not only that the 3p25 breakpoint lies within the PPAR*γ* gene, but also that the 2q13 breakpoint lies within the PAX8 gene. Indeed, the translocation brings together these two genes to form a neogene, which expresses a fusion protein (*PAX*8/PPAR*γ* fusion protein, designated PPFP) [3].

**Figure 1:**
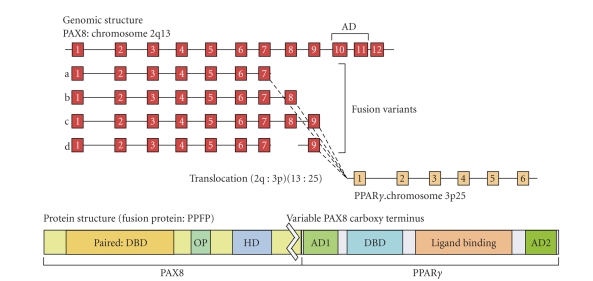
PAX8/PPAR*γ* rearrangement illustrating the genomic structure with exon arrangement and sites of fusion. The PAX8 activation domain (AD) is eliminated in all fusion events. The protein structure of the predicted fusion protein is shown and contains the PAX8-paired domain, containing the DNA binding domain (DBD), the octapeptide motif (OP), and the truncated homeodomain (HD). All of the functional domains of PPAR*γ* gene, including activation domains 1 and 2 (AD1 and AD2), DBD, and ligand binding domain are retained in the fusion protein.

Native PAX8 is a transcription factor important in thyroid follicular cell differentiation and in the regulation of a number of thyroid-specific genes [36]. The chromosomal translocation described by Kroll fuses the promoter and proximal 5’ coding sequence of PAX8 inframe with a nearly full-length PPAR*γ*, resulting in the production of the fusion protein PPFP, whose expression is under the transcriptional regulation of the PAX8 promoter [3]. Several splice variants have been identified for PPFP, which appear to be frequently coexpressed [3, 36, 37]. Variants described to date are shown in Figure 1. In each case these PAX8, fragments are fused to PPAR*γ* exons 1 to 6 [3, 36, 37]. All of the known variants include the paired and partial homeobox DNA binding domains of PAX8, as well as the DNA binding, ligand binding, RXR dimerization and transactivation domains of PPAR*γ*-1 [3]. These attributes make it likely that PPFP will retain at least some of the DNA and ligand binding properties of each of the native transcription factors, with the potential for significant impact on either the PAX8- or PPAR*γ*-mediated pathways, or both.

A number of studies, using RT-PCR, nested PCR, FISH, or Western analysis, have confirmed the relatively high prevalence of the PAX8/PPAR*γ* rearrangement or expression of PPFP in follicular thyroid lesions, though the precise incidence varies by cell type and method of detection (Table 1). FTCs have the highest incidence at 36% (range 0–63%) with FAs exhibiting lower rates of around 11% (range 0–55%). Only one case of HCC has shown the PAX8/PPAR*γ* rearrangement. PPFP does not appear to be expressed in classical PTC, but karyotyping has shown at (2;3)(q13;p25) in one case of follicular variant of papillary thyroid cancer (FVP) [49] and intense PPAR*γ* immunostaining was seen in three PPFP-negative FVP [50], perhaps arguing for an alternative mechanism of PPAR*γ* overexpression in these tumors. Although Castro et al. [44, 47] found that 37–50% of FVP were positive for PPFP by RT-PCR and FISH, three other studies have found no such rearrangements [36, 38, 39]. It is, nevertheless, tempting to speculate whether those few cases of FVP that develop lung metastasis [51] or display an encapsulated growth pattern [52] might behave with a phenotype more reminiscent of a follicular carcinoma than papillary because of PPFP expression. Larger studies are needed to determine the frequency of expression of PPFP in this tumor variant, and to assess the possible coexpression of genes known to be associated with PTC, such as RAS and BRAF mutations and RET/PTC rearrangements.

## 4. PAX8/PPAR*γ* AND PPAR*γ* FUNCTION

To further investigate the function of PAX8/PPAR*γ*−1, Kroll evaluated PPAR*γ* response element (PPRE) activity in the presence of the fusion gene and wild-type PPAR*γ* in a PPAR*γ*-null osteosarcoma U2OS cell line [3]. In contrast to the wild-type PPAR*γ* gene, the fusion gene was ineffective in stimulating ligand-induced gene expression. Furthermore, the coexpression of the fusion gene with wild-type PPAR*γ* abrogated the PPAR*γ*-mediated gene expression, in an apparently dominant negative fashion [3].

In vitro studies using an immortalized human thyroid cell line have shown accelerated growth in cells transiently transfected with PPFP compared to wild-type PPAR*γ* or vector only [53]. Increased proliferation was confirmed with cell cycle transit studies, which showed a lower proportion of PPFP-transfected cells in the resting-phase (G_0_/G_1_) compared to vector and diminished rates of apoptosis in the PPFP-positive cells. Similar results were seen with stable transfection experiments, in which cells stably expressing PPFP demonstrated a growth advantage over vector-transformed cells. These data are consistent with the hypothesis that PPFP, acting as a dominant negative inhibitor of wild-type PPAR*γ*, inhibits the normal tumor suppressor mechanism of PPAR*γ*, and consequently acts as an oncogene. Furthermore, PPFP stable cell lines showed improved colony-formation on soft agar, a characteristic associated with malignant transformation [53].

Consistent with Kroll's results, cotransfection of PPFP and wild-type PPAR*γ* in immortalized human thyroid cells led to a significant decline in PPAR*γ* transactivation [53]. In these studies, GW9662, a potent PPAR*γ* inhibitor, demonstrated a dose-dependent increase in cell growth of vector-transfected cells, but did not increase growth further in the PPFP-transfected cells. Similar effects were observed upon expression of a dominant negative PPAR*γ* mutant in these cells. Loss of contact inhibition and anchorage dependence, which also correlate with malignant transformation, were also observed upon overexpression of PPFP. The studies of Powell et al. [53] provided the first direct evidence for the oncogenic potential of the PAX8/PPAR*γ* fusion gene, confirming increased proliferation, decreased apoptosis, and a dominant negative effect of PPFP on wild-type PPAR*γ*.

The influence of PPFP on PPRE-dependent transcription appears to be cell line-dependent. Au et al. [54] demonstrated that the fusion gene not only had a dominant negative effect on PPRE expression in HeLa cells, but also stimulated the expression of the PPRE-dependent promoter in a PPAR*γ* ligand-dependent manner in FRTL-5 and Nthy-ori cells, immortalized rat and human thyroid cell lines, respectively. These differences might be related to differences in the ways these cells have been immortalized. Thus HeLa cells utilized the HPV E6 gene and Nthy-ori cells were immortalized with the SV40 large T antigen, whereas FRTL5 cells are a continuous line of functional, nontransformed rat thyroid cells that depend on thyroid-stimulating hormone (TSH) for sustained growth. Whatever the mechanism, the results of Au et al. [54] are in direct contradiction to Powell's findings, in which PPAR*γ* agonists did not augment the PPFP response [53]. The reasons for this discrepancy are not known. Nevertheless, PPFP expression in FRTL-5 cells showed increased proliferation by ^3^H-thymidine incorporation and soft agar assays [54], findings that are fully consistent with Powell's data [53]. Although further study is clearly warranted to clarify the mechanism of PPFP action in the cell lines, the data indicate that PPFP can act as a dominant negative inhibitor as well as an independent ligand-responsive transcription factor in a promoter-dependent manner.

It is still not clear whether PPFP alone is sufficient to promote tumorigenesis, or whether additional genetic events are a prerequisite for this fusion gene to exhibit an oncogenic impact. One strong candidate, RAS gene mutations, which are seen in up to 50% of follicular tumors, rarely occur within the same tumor as PAX8/PPAR*γ* rearrangements, suggesting that these putative oncogenes form two distinct pathways of carcinogenesis [43]. For each of these pathways, an additional step or series of steps may be required before the development of the full malignant phenotype.

## 5. PAX8/PPAR*γ* AND PAX8 FUNCTION

Relatively few studies have assessed the impact of PAX8/PPAR*γ* rearrangements on wild-type PAX8 function. Au et al. [54] and Espadinha et al. [55] evaluated the impact of PAX8/PPAR*γ* on genes containing PAX8 response elements: sodium-iodine symporter (NIS), thyroid peroxidase (TPO), thyroid stimulating hormone receptor (TSHR), and thyroglobulin (Tg). Each of these promoters is regulated by PAX8, while the Tg promoter is regulated by both PAX8 and thyroid transcription factor-1 (TTF1), which exhibit a synergistic effect when both promoters are combined in vitro. In human thyroid cancer cell lines, PPFP expression resulted in a complex mixture of stimulatory and inhibitory effects on PAX8-responsive genes, including in PPAR*γ* ligand-dependent and -independent effects. NIS gene expression was stimulated in response to PPFP expression alone in one study [54], although this apparently stimulatory effect required cotransfection of PPFP with wild-type PAX8 in another study [55]. TPO transcription was also increased by PPFP [54], while TSHr expression was inhibited [55]. Repression of the Tg promoter was also seen in response to PPFP [54], but again one study found that cotransfection with PPFP and PAX8 was necessary for this inhibitory effect to be seen [55]. However, in both studies, the fusion gene inhibited PAX8-mediated transcription of Tg in a dominant-negative fashion [54, 55], while the addition of ciglitazone did not reverse this dominant negative effect [54].

Consequently, the effects of the PAX8-PPAR*γ* gene translocation on PAX8 function seem to be complex. TSHr and Tg expressions, both of which are associated with highly differentiated thyroid tissue, are downregulated by PPFP, though it is not clear whether the reduced expression of these genes truly alters cell differentiation status in vivo. In contrast, NIS and TPO expression are enhanced by the expression of PPFP, although once again the impact on cell function is not known. Whether any of these findings relates directly to the oncogenic actions of PPFP, and consequently the impact on the behavior and biology of human FTC, remains to be determined.

## 6. PAX8/PPAR*γ* REARRANGEMENTS: BENCH TO BEDSIDE

On the basis of the data discussed above, PPFP appears to be important in the development of at least a subset of thyroid follicular neoplasms, and has, therefore, been proposed as a possible oncogene in follicular thyroid carcinoma. The most obvious direct clinical utility of this discovery is the possibility that PPFP status could provide a presurgical test of malignancy within the troublesome group of biopsy specimens currently described as “suspicious for follicular neoplasm.” These lesions represent approximately 20% of all fine needles aspiration biopsies of thyroid nodules, and create a diagnostic challenge because the minority (10–15%) that prove ultimately to be malignant cannot currently be distinguished by cytological criteria from those that prove to be benign. Consequently, the recommendation for all such patients would be to undergo surgery [56], something that might be avoided with a preoperative test of sufficient accuracy. Unfortunately, the finding of a subset of PPFP-positive adenomas reduces the negative predictive value of a preoperative PPFP biopsy finding to 47.4%, while the presence of PPFP in a minority of FTC reduces the positive predictive value to 80.6% (Table 1).

RT-PCR, real-time RT-PCR, and FISH have all been used experimentally to detect translocations, though none is yet proven in a prospective, clinical setting [3, 36, 39, 42]. PCR, probably the technique most easily adapted to a rapid turnover, clinical setting, is concordant with PPAR*γ* immunohistochemistry in up to 80% of cases [36]. When the definition of “positive” PPFP is restricted to only strong, diffuse staining, immunohistochemistry concordance improves to 100% in some studies [7, 39], and it may be that RT-PCR techniques will actually prove superior to the immunohistochemistry “gold standard” for the detection of the fusion event (Algeciras-Schimnich, A. and Grebe, S.K.G., unpublished data). The possibility of false-positive immunostaining is real since normal thyroid tissue, chronic lymphocytic thyoiditis, or benign tissue adjacent to malignancies may show moderate to strong nuclear staining for PPAR*γ* expression. [39, 57], so the RT-PCR-based approach may prove to have a better negative predictive value. There are a number of possible explanations for this apparently “false-positive” staining, including alternate PAX8/PPAR*γ* breakpoints, 3p25 aneusomy, overexpression of wild-type PPAR*γ*, or rearrangements involving PPAR*γ* and a non-PAX8 partner [57].

Despite these challenges, Sahin et al. demonstrated that a reliable preoperative assay for PPFP might improve the accuracy of intraoperative frozen section by significantly reducing the false-negative rate of this technique, and therefore reducing the need for second (completion) surgeries for patients with follicular carcinoma who undergo primary thyroid lobectomy [42]. Immunohistochemistry formed the basis for this retrospective study, so clinical implementation would require confirmation with a more practical preoperative or intraoperative technique (most likely RT-PCR). In this archival tissue analysis, however, the sensitivity was improved from 85% with frozen section alone to 97% with the combination of frozen section and PPAR*γ* status. Several false-positive PPAR*γ* staining results led to a positive predictive value of only 72%, but the overall negative predictive value of frozen section plus immunostaining at this institution was 99%, meaning that five additional cases of carcinoma in this series of 39 cancers could have been identified intraoperatively, reducing the need for completion thyroidectomy to a single patient (3%).

Currently, there is no evidence that PPFP status predicts outcome in follicular thyroid cancer, with no correlation with proven predictive factors of gender, age, regional nodal spread, or tumor size [28, 39, 42]. The same is true for PPAR*γ* aneusomy or other PPAR*γ* rearrangements found in follicular cancers, which is not correlated with TNM stage [7]. However, patients with PPAR*γ* rearrangements may have a higher prevalence of previous nonthyroid cancers [7] and PPFP rearrangements may be associated with an increased incidence of multifocal capsular invasion or vascular invasion [39], although all of these findings remain in dispute [28, 42]. Larger and more comprehensive outcome analysis will be necessary to resolve these differences in the findings of multiple small studies.

## 7. PPAR*γ* AGONIST THERAPY IN THYROID CANCER

Follicular cell-derived thyroid cancers carry a generally good prognosis. Standard therapy involves near-total resection of the thyroid with adjuvant ^131^I radioablation of remnant thyroid tissue in most cases. A subset of tumors exhibits a more aggressive clinical course, and may show features of dedifferentiation, which has been associated with decreased expression of thyroid-specific genes such as Tg, TPO, NIS, and TSHR [15, 58]. These dedifferentiated thyroid cancers may consequently lose their ability to accumulate and concentrate radioiodine, making these tumors unresponsive to further ^131^I therapy. Traditionally, patients with iodine-insensitive tumors have had few therapeutic options and further basic and applied research is needed to identify suitable therapeutic targets for treatment of these patients; PPAR*γ* provides one such target.

Thiazolidinediones, including troglitazone, rosiglitazone, and pioglitazone, are PPAR*γ* agonists used in the treatment of type 2 diabetes. These and other PPAR*γ* agonists have been investigated in vitro in various cancer cell lines with evidence of growth inhibition and tumor cell apoptosis [18–23, 59]. Small clinical trials in liposarcoma and prostate cancer, which exhibit PPAR*γ* expression, have also been promising in these malignancies, which exhibit PPAR*γ* expression [60, 61]. Recent in vitro evaluations in thyroid cancer cell lines have hinted at a possible role for PPAR*γ* agonist therapy in redifferentiating neoplastic tissue, potentially enhancing the response to currently available therapies [62–67]. Expression of CD97, a marker of cell dedifferentiation, decreased in one follicular carcinoma cell line after therapy with troglitazone [16]. In the same experiment, expression of NIS, a gene associated with well-differentiated thyroid tissue, whose protein product is responsible for iodine concentration within thyroid cells, increased with agonist treatment compared to control in both a papillary and follicular carcinoma cell lines. Such an approach of “redifferentiation” might open up the possibility to restore radioactive iodine sensitivity in some tumors. Anaplastic cancer cell lines have been studied in similar experiments, and expressions of Tg, TSHR, NIS, and TPO were all increased after treatment with rosiglitazone [12]. These findings imply that PPAR*γ* agonists may prove to be an effective therapy for improving response to ^131^I radiotherapy, even in patients without known PAX8/PPAR*γ* rearrangements.

To date, three reports have been published assessing rosiglitazone therapy in patients with recurrent thyroid cancer as indicated by elevated Tg levels, but negative pretreatment whole body iodine scans (Tg-positive, scan-negative thyroid cancer). Philips et al. [68] treated 2 follicular cancer and 3 papillary cancer patients with rosiglitazone 4 mg daily for one month and then 8 mg daily for three months. Whole body ^131^I scanning (WBS) using recombinant human TSH (rh-TSH) was negative for all 5 patients at the onset of the study. Basal Tg levels rose in 3 patients while the rhTSH-stimulated Tg increased in two of the 3 patients after rosiglitazone therapy. Posttreatment rhTSH-stimulated WBS was faintly positive in one patient. Elias and Lizotte [69] reported a single case of papillary thyroid cancer in which rosiglitazone caused a marked increase in ^131^I uptake on WBS. Following treatment with 250 mCi ^131^I, the WBS became negative, suggesting that the cancer had been effectively treated. Kebebew et al. [70] have reported the largest number of patients to date treated with a thiazolidenedione: 8 PTC, 1 FVP, and 1 FTC. These patients received rosiglitazone 4 mg daily for 7 days, then 8 mg daily for 49 days. Four patients (40%) had conversion from negative to positive WBS after rosiglitazone, suggesting a possible redifferentiation effect. At 6 months of followup, 2 patients had improved Tg levels (one follicular and one FVP patient), 3 had stable levels (all papillary cancer patients), and 5 patients had increased Tg levels compared to baseline. After 11 months of followup, 4 patients had a partial response to rosiglitazone, exhibiting decreased Tg levels or increased ^131^I uptake, 4 had stable disease, and 2 had progression of disease as indicated by increased Tg levels. In all cases, rosiglitazone was apparently well tolerated with no significant adverse events. Overall, 6 of 16 patients (5 papillary, 1 FVP) showed uptake on WBS after rosiglitazone therapy, indicating that a subset of patients may have experienced redifferentiation of their cancers. Whole body iodine scanning did not always correlate with Tg levels and caution should be exercised in interpreting Tg levels in these studies because an increase in Tg could indicate improved differentiation, rather than being a sign of increasing tumor mass. Only one patient in this study received additional ^131^I radiotherapy, although therapy was apparently successful, with a low Tg post-radiotherapy. Larger studies with longer followup are needed to see if thyroid cancers treated with PPAR*γ* agonists show improved response to ^131^I radioablation or decreased mortality, the truly important clinical outcome.

Very little data is currently available on combination therapy with PPAR*γ* and other chemotherapeutic agents. Aiello et al. [12] evaluated the in vitro response of rosiglitazone plus doxyrubicin, a standard agent used in anaplastic thyroid cancer, in ATC cell lines. They found a markedly increased effect of doxyrubicin in PPAR*γ*-positive cell lines when combined with rosiglitazone, but no effect in a PPAR*γ*-negative cell line. Copland et al. [71] combined paclitaxel with a novel PPAR*γ* agonist, RS5444, in a variety of ATC cell lines. This combination demonstrated synergistic effects on inhibition of proliferation and stimulation of apoptosis in vitro. When athymic nude mice were implanted with the responsive ATC cell lines, tumor growth was inhibited by monotherapy with RS5444. No clinical studies or case reports have yet addressed combination therapy in humans with thyroid cancer, though a clinical trial is currently under development by our group.

## 8. CONCLUSION

Emerging genetic and molecular information acquired over the last 2 decades has begun to unravel the pathogenesis of thyroid cancer and in the future may open the door to potential novel therapies for patients with previously untreatable disease. Research focusing on PPAR*γ* in a variety of cancer cell lines has implied a tumor suppressor function for wild-type PPAR*γ*, while PPAR*γ* downregulation or inhibition may be one factor in the development of at least some thyroid cancer types.

Chromosomal alterations of PPAR*γ*, resulting in the expression of the fusion protein PPFP, may be an early event in the development or progression of follicular thyroid cancer and perhaps the follicular variant of papillary cancer. The detection of these alterations in FAs may support a stepwise adenoma to carcinoma sequence, or indicate the presence of “carcinoma in situ.” However, the PAX8/PPAR*γ* rearrangement in itself may not be sufficient for the development of a malignant phenotype: additional genetic or epigenetic events may be required to enable the full phenotypic expression of follicular thyroid carcinoma.

Several lines of data suggest that PPFP, either through PPAR*γ* inhibition, PAX8-dependent gene expression modulation, or both, leads to downstream effects, which are at least in part mediated by the NF*κ*B pathway. These altered pathways stimulate cell proliferation and inhibit apoptosis, but there may be several other paths through which PPFP modulates the tumor phenotype, including alteration of cell differentiation status and expression of the sodium iodide transporter NIS, which may have an impact on the efficacy of our current therapeutic options.

PPAR*γ* represents an attractive therapeutic target in a variety of thyroid cancers, including anaplastic, follicular, and papillary thyroid cancers. Although in vitro data is promising, early studies using PPAR*γ* agonists to treat iodine-insensitive recurrent thyroid cancer are promising, but inconclusive so far. Larger studies with longer followup will be needed to clarify the potential for PPAR*γ* agonists to act as “redifferentiation” agents, Nevertheless, the availability of a number of approved, orally administered, well-tolerated agents makes this group of drugs an attractive option for study.

## Figures and Tables

**Table 1:** Occurrence of PAX8/PPAR*γ* rearrangements in differentiated thyroid carcinomas.

Author	Method	Benign nodular hyperplasia	Follicular adenoma	Follicular carcinoma	Hürthle cell carcinoma	Papillary carcinoma	Follicular variant of papillary	Anaplastic carcinoma
Kroll [3]	RT-PCR	0/10	0/20	5/8 (63%)	—	0/10	—	—
Martelli [14]	RT-PCR	—	—	0/5	—	0/41	—	0/5
Zhu [38]	RT-PCR	—	—	—	—	0/46	0/30	—
Nikiforova [39]	RT-PCR and nested PCR	0/16	2/25 (8%)	8/15 (53%)	0/12	0/23	0/12	0/2
Marques [36]	RT-PCR and nested PCR	0/2	2/16 (13%)	5/9 (56%)	—		0/9	0/4
Aldred [29]	RT-PCR	—	—	2/19 (11%)	—	—	—	—
Lacroix [40]	RT-PCR	—	1/16 (6%)	4/26 (15%)	0/4	—	—	0/5
Hibi [41]	RT-PCR	0/12	0/12	0/6	—	0/12	—	—
Marques [28]	RT-PCR and FISH	0/28	6/36 (17%)	6/24 (25%)	—	0/38	—	—
Sahin [42]	RT-PCR	—	4/31 (13%)	31/54 (57%)	1/23 (4%)	—	—	—
Nikiforova [43]	RT-PCR and FISH	—	1/23 (4%)	13/33 (36%)	0/19	—	—	—
Castro [44]	RT-PCR and FISH	—	—	—	—	—	4/8 (50%)	—
Cheung [37]	RT-PCR	—	6/11 (55%)	6/17 (35%)	—	—	—	—
Foukakis [45]	RT-PCR	—	1/8 (13%)	5/25 (20%)	—	—	—	—
Dwight [46]	RT-PCR, FISH and Western	—	1/40 (3%)	10/34 (29%)	—	—	—	0/13
Castro [47]	RT-PCR and FISH	—	3/9 (33.3%)	10/22 (45.5%)	—	0/2	9/24 (37.5%)	—
Giordano [48]	RT-PCR	—	—	7/13 (54%)	—	—	—	—

TOTAL		0/68	27/247 (11%)	112/310 (36%)	1/58 (2%)	0/172	13/83 (16%)	0/29
